# Upgraded Kalman Filtering of Cutting Forces in Milling

**DOI:** 10.3390/s20185397

**Published:** 2020-09-21

**Authors:** Giovanni Totis, Zoltan Dombovari, Marco Sortino

**Affiliations:** 1 Polytechnic Department of Engineering and Architecture, University of Udine, 33100 Udine, Italy; 2 MTA-BME Lendület Machine Tool Vibration Research Group, Department of Applied Mechanics, Budapest University of Technology and Economics, H-1521 Budapest, Hungary; dombovari@mm.bme.hu

**Keywords:** milling, cutting forces, dynamometer, piezoelectric, dynamics, compensation, Kalman filter

## Abstract

Advanced piezoelectric dynamometers with a wide frequency bandwidth are required for cutting force measurement in high-speed milling and micromilling applications. In many applications, the signal bandwidth is limited by the dynamic response of the mechanical system, thus compensation techniques are necessary. The most effective compensation techniques for a full 3D force correction require an accurate and complex identification phase. Extended Kalman filtering is a better alternative for input force estimation in the presence of unknown dynamic disturbances. The maximum bandwidth that can be currently achievable by Kalman filtering is approximately 2 kHz, due to crosstalk disturbances and complex dynamometer’s dynamics. In this work, a novel upgraded Kalman filter based on a more general model of dynamometer dynamics is conceived, by also taking into account the influence of the force application point. By so doing, it was possible to extend the frequency bandwidth of the device up to more than 5 kHz along the main directions and up to more than 3 kHz along the transverse directions, outperforming state-of-the-art methods based on Kalman filtering.

## 1. Introduction

Measuring the cutting forces during milling operations plays a fundamental role in the development of advanced cutting tools that should enhance the cutting process performance. For this purpose, special dynamometers having a wide frequency bandwidth are required [1]. This is even more critical in modern applications, where the use of small cutter diameters at high cutting speeds is not restricted to micromilling applications. Moreover, cutting force sensors will probably be integrated into next-generation machine tools. Together with other additional sensors, they will allow the effective application of in-process advanced control strategies based on the digital twin concept [2].

Nevertheless, state-of-the-art devices for cutting force measurement in milling are typically affected by several issues that drastically limit their quasi-static and dynamic performances.

Quasi-static disturbances mostly derive from structural nonlinearities and crosstalks as well as from the sensitivity to temperature or other uncontrolled environmental factors. For example, differential thermal expansions of the machine tool elements embedding the force sensors may occur because of cutting process heat or spindle rotation, as illustrated in [3]. As a result, they may alter sensors’ preload (when using piezoelectric load cells), thus causing low-frequency drifts in the signals. Quasi-static disturbances may also derive from gravitational and inertial effects such as those arising during the controlled movements of the workpiece—plateform dynamomter—rotary table system of a five axes CNC milling machine, as showed by Klocke et al. [4]. Needless to say, quasi-static disturbances need to be corrected for many practical applications based on cutting force measurement.

On the other side, state-of-the-art systems for cutting force measurement in milling are affected by a moderate frequency bandwidth, because of the following reasons,

- sensitivity issues such as those affecting dynamometers based on strain gauges [5], capacitance sensors [6], optical sensors [7,8,9] and other sensors requiring large structural deformations at the inspected point in order to achieve a satisfactory signal to noise ratio; this is typically obtained by weakening the machining system, thus reducing the resonance frequency;
- load effects such as those introduced by rotating dynamometers [10,11] that may drastically reduce the first resonance frequency of the machine tool spindle (below 500 Hz) with respect to the unaltered configuration where no sensor (thus neither additional modal mass nor additional flexibility) is integrated into the machining system; and
- dynamic inertial disturbances deriving from the undesired but unavoidable oscillations of the modal mass placed in front of the piezoelectric load cells in case of plateform dynamometers; by so doing, even low-frequency vibration modes of the machine tool table where the dynamometer is clamped may cause undesired force fluctuations that cannot be avoided, as explained in [12].

The moderate frequency bandwidth of modern dynamometers could be extended in three ways:

- by improving the device hardware, i.e., by selecting sensors and electromechanical components having higher performances;
- by improving the mechanical design of the device, e.g., by reducing the modal mass in front of the force sensors as described in [12,13,14,15]; and
- by applying advanced filtering techniques to the measured signals, which may considerably extend the frequency bandwidth above the structural limits [16].

An interesting prototype of a sensorized clamping vise incorporating both strain gauges and piezolectric sensors has been recently developed by Rezvani et al. [17] with the aim of accurately measuring both the static force components (mainly due to clamping) and the dynamic forces (due to the cutting process) acting on the workpiece. Nevertheless, the final frequency bandwidth was still limited.

Similarly, Luo et al. [18] used last generation PVDF piezo-films as a new alternative to other more conventional sensors for enhancing the dynamic performance of a plateform dynamomter. However, only some preliminary and partial results regarding the achieved frequency bandwidth were reported there.

Nevertheless, improving device hardware and design has proven not to be sufficient to considerably enhance its frequency bandwidth as required by many current applications.

Therefore, adequate filtering techniques should be applied whenever possible to compensate both quasi-static and dynamic disturbances affecting every kind of dynamometer.

The most effective approaches are basically twofold: Optimal Inverse Filtering and Augmented (or Expanded) Kalman Filtering. Optimal Inverse Filtering is based on a robust inversion of the 3×3 global transmissibility matrix describing the dynamic connection between the resultant input force and the measured resultant. First attempts in this direction were made by Castro et al. in 2006 [19] and by Girardin et al. in 2010 [20], who recognized the importance of both direct and cross-transmissibility functions when trying to obtain a full 3D force correction. Although their identification was good up to 16 kHz, the proposed correction method was effective up to only 2 kHz. The method was considerably refined by Wan et al. [21] and especially by Korkmaz et al. [16], who achieved an impressive dynamic compensation up to 25 kHz for a micromilling application. An interesting application of the Optimal Inverse Filter method was recently proposed by Kiran in 2019 [22] for cutting force correction in the presence of flexible workpieces. However, this technique is feasible only when all the coherence functions associated to the 3 × 3 Transmissibility Frequency Response Functions (TFRFs) are close to unity within the whole frequency bandwidth under interest. This requires a very accurate and complex identification procedure and an advanced modal equipment [16,23].

On the contrary, when the TFRFs are contaminated by noise or in the presence of other unknown dynamic disturbances the Augmented Kalman Filter (AKF) technique should be preferred. The compensated TFRFs will be almost flat up to a higher frequency, though it will be affected by a linear phase decay. Another advantage is that Kalman filtering can be applied for real-time applications, whereas the Optimal Inverse Filtering is not adequate for this purpose.

In their pioneering work, Albrecht et al. [24] implemented an Augmented Kalman Filter for one-dimensional force compensation. Chae et al. [25] included the acceleration signals for a better compensation of the inertial disturbances, thus reaching about 5 kHz of usable frequency bandwidth. Nevertheless, their approach was still one-dimensional. A very accurate model of dynamometer’s TFRF is necessary for an effective application of the AKF approach, as it was proved by Scippa et al. [26]. Moreover, in this work the cutting force filtering was one-dimensional.

Very recently Corrigan et al. [27] presented two new methods for compensating the dynamic disturbances of cutting forces in milling: Regularized Deconvolution (RD) and Sliding Mode Observer (SMO). These innovative methods do not require any prior knowledge of process or measurements noise, which is a great advantage with respect to the classic AKF. Nevertheless, the effects of crosstalks and position-dependent dynamometer dynamics were not treated there.

The aim of this work is to provide a novel methodology for a full 3D force compensation by using the Augmented Kalman Filter approach. This result will be obtained by taking into account the full, complex dynamic behavior of the device along direct and cross directions and by also considering the influence of the force excitation point for any fixed direction. Singular Value Decomposition (SVD) will be largely and repeatedly applied for the development of a minimal dynamic model which is the basis of the Upgraded Augmented Kalman Filter (UAKF) described here.

In the next section, the results of the experimental modal analysis will be shown. Afterwards, the main algorithm for dynamometer dynamics identification and filtering will be described. The filter performance will be evaluated by using data from pulse tests as well as from real cutting tests. Conclusions will be drawn in the final section.

## 2. Experimental Investigation on Dynamometer Dynamics

In this section, the complex dynamic behavior of the dynamometer will be showed, and its dependence on the force application point will be revealed. Then, a novel and more general dynamic model will be conceived that will become the foundation for the Upgraded Augmented Kalman Filter proposed here. Eventually, the algorithm for model identification and Kalman filter determination will be outlined.

### 2.1. Experimental Setup

**Figure 1:**
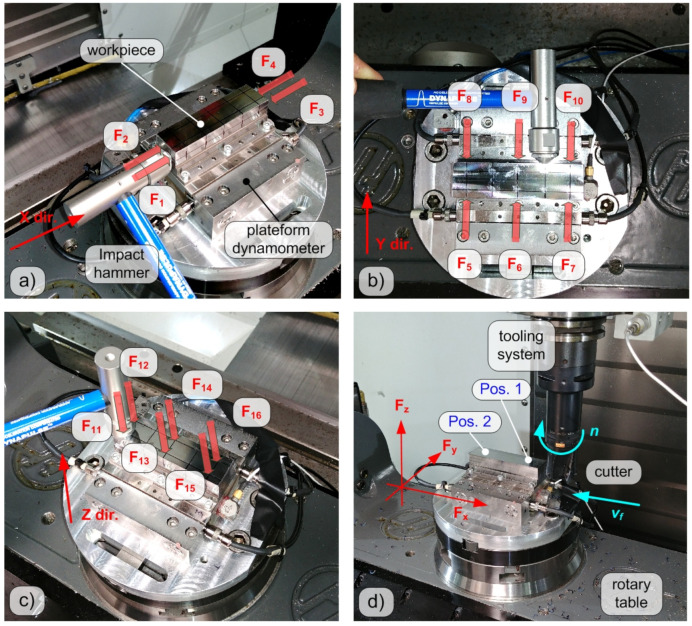
Impulsive forces denoted by $F_{p}$ with p=1,…,4 were applied at 4 different locations along the *X* direction (**a**). Impulsive forces denoted by $F_{p}$ with p=5,…,10 were applied at 6 different locations along the *Y* direction (**b**). Impulsive forces denoted by $F_{p}$ with p=11,…,16 were applied at 6 different locations along the *Z* direction (**c**). The experimental set-up for the final cutting tests (**d**).

Experimental modal tests were carried out at LAMA FVG (Laboratory for Advanced Mechatronics of the University of Udine that is located in Udine, Italy) on a 5-axes CNC milling machine (Haas VF2-TR), see Figure 1d. All sensor signals were sampled at 51.2 kHz by a National Instruments Data Acquisition device (cDAQ-9178 with NI9215 modules) and stored on a PC for further analysis, which was carried out in the MathWorks MATLAB environment.

A self-made dynamometer was previously constructed embedding four triaxial high-sensitivity piezoelectric force cells Kistler 9016B4, similarly to the Kistler Minidyn 9256C2 architecture. The central plateform was made of 316L stainless steel (about 390 g) while the lateral elements connecting the cells to the rotary table of the machine tool were made of Ck45 steel. A Ck45 carbon steel workpiece having a mass of about 430 g and a hardness of about 198 HB was clamped on the central plateform by using six M3 screws. Pulse testing technique was applied along the three main directions of the device by using impact hammer type Dytran 5800B4 (sensitivity 2.41 mV/N).

### 2.2. Preliminary Results from Modal Analysis

According to the classical calibration procedure, the dynamometer (dyn) should be loaded along different directions in order to derive the entire map between the (quasi-static) applied input $R_{k}$ and the sensed output $R_{\mathrm{dyn},i}$ components (where i,k=x,y,z) of the resultant force R. For any given direction, the device should also be loaded at different points representing the possible force localizations under the real sensor application. In the current case, a total of *P* = 16 loading points were considered: 4 along the *X* direction (Figure 1a), 6 along the *Y* direction (Figure 1b) and 6 along the *Z* direction (Figure 1c). A similar procedure was also applied in [28] but with a different purpose, i.e., for applying RCSA and TPA techniques to predict the combined dynamics of the workpiece–dynamometer system. Consequently, in the current case, the components of the resultant (applied) input force are given by the sums

(1) $$\left\{\begin{array}{c} R_{x}=\sum_{p=1}^{4}F_{p},\\ R_{y}=\sum_{p=5}^{10}F_{p},\\ R_{z}=\sum_{p=11}^{16}F_{p}. \end{array}\right.$$

According to the classic approach, the components of the resultant should be estimated as linear combinations of the available (sensed) output signals $F_{\mathrm{dyn},q}$, i.e.,

(2) $$R_{\mathrm{dyn},i}=\sum_{q=1}^{Q}\psi_{iq}F_{\mathrm{dyn},q}\approx R_{i},$$

where i=x,y,z. Here, Q=12 output signals are derived from the 4 triaxial load cells composing the device. The static calibration coefficients $\psi_{iq}$ are typically obtained from a linear regression procedure where many quasi-static force inputs are compared to quasi-static force outputs, by varying the excitation direction, the force application point, and the force intensity. The low-frequency contents of the hammer impulses and of the corresponding output transients – measured during the modal analysis – were used for this purpose, by achieving a very good interpolation between quasi-static inputs and outputs (squared linear correlation coefficient $r^{2}=0.997$).

The dynamic relation between input and outputs is represented by the Transmissibility Frequency Response Functions (TFRFs)

(3) $$T_{ik}\left(j\omega \right)=\frac{R_{\mathrm{dyn},i}\left(j\omega \right)}{R_{k}\left(j\omega \right)},\;\;\;\;\;\;\;\;i,k=x,y,z.$$

**Figure 2:**
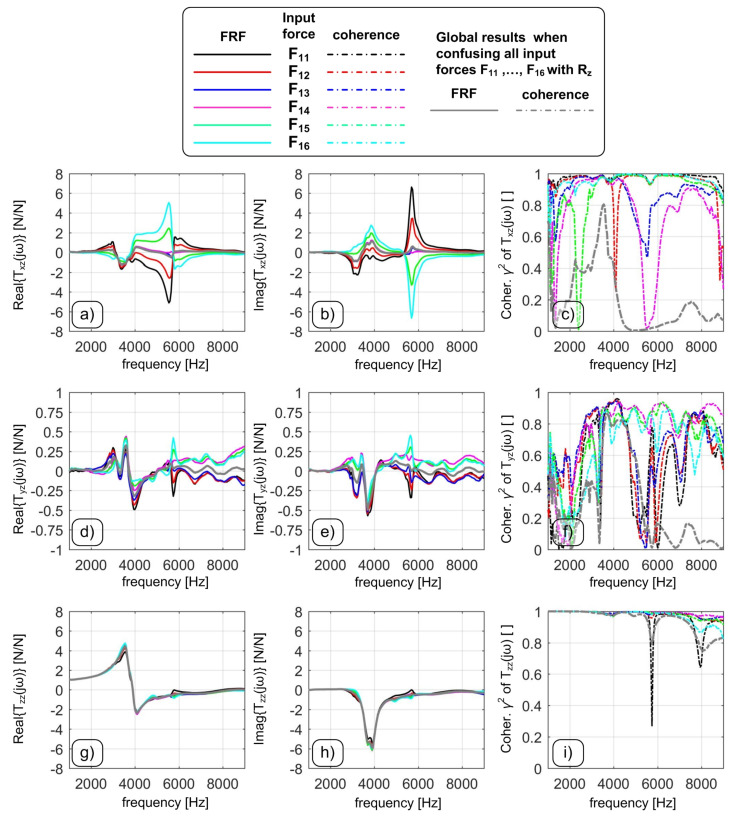
The real part, imaginary part and coherence function of the cross TFRF $T_{xz}$ are illustrated in (**a**), (**b**) and (**c**), respectively. Different colors refer to different input force conditions $F_{p}$, though all along the *Z* direction. Similarly the real part, imaginary part and coherence function of the cross TFRF $T_{yz}$ are illustrated in (**d**), (**e**) and (**f**), respectively. Eventually, the real part, imaginary part and coherence function of the direct TFRF $T_{zz}$ are illustrated in (**g**), (**h**) and (**i**), respectively.

For a fixed input direction, the obtained transmissibilities should be ideally independent from the force application point. Nevertheless, this is not true as it was already pointed out experimentally by Korkmaz in 2014 [23]. It is worth noting that this fact can be ignored only in micromilling, where the milling operation can be restricted to a very small area above the measuring device, as it was the case studied in [16,23]. On the contrary, in conventional milling it is not possible to make such assumption and the influence of the tool-workpiece contact point on cutting forces cannot be neglected in the middle–high frequency domain. This is clear from Figure 2, where the cross TFRF $T_{xz}$ is particularly sensitive to the selected location where the impulsive forces $F_{p}$ are applied.

**Figure 3:**
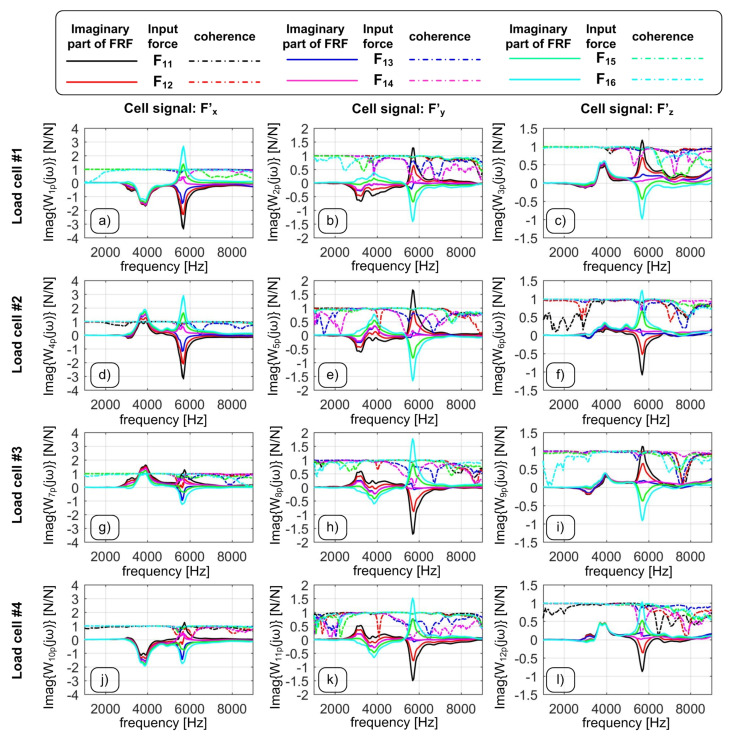
Imaginary parts and coherences of the TFRFs derived from the 12 output signals when the system was hit in the *Z* direction at the different points of Figure 1c. $F_{k}^{\prime }$ are the load cell signals in the cell reference frame. From (**a**) to (**l**), all the TFRFs $W_{qp}$ with q=1,…,12 and p=11,…,16 (i.e., with inputs along the *Z* direction) are represented.

If all the transients corresponding to different inputs (all along *Z* but at different target points) are elaborated at the same time, a kind of weighted, average result is obtained (gray lines). However, such average is affected by a low coherence even where the TFRFs (derived from a single target point) are reliable. For example, at about 3.5 kHz all coherence functions are above 90% but the coherence function of the global TFRF is low. Under these circumstances the Optimal Inverse Filtering technique presented in [16] cannot be applied. The reason is that different excitation points do stimulate the device eigenmodes differently [23]. This is also visible in Figure 3, where the TFRFs of each load cell signal are shown. In the frequency interval from 2.5 to 7 kHz there is a strong dependence on the input location, that is not canceled by the linear combination of Equation (2) because the system is not perfectly symmetric and it is not loaded in a perfectly symmetric, ideal way. In other words, the static and dynamic sensitivity of each output signal depends on both the input force direction and the target point, thus the formulation (2) cannot represent a correct solution in a wide frequency interval. Thus, a more general approach is needed.

## 3. The Upgraded Augmented Kalman Filter (UAKF)

In order to achieve an effective 3D force correction, the resultant force components $R_{k}$ have to be derived from the output signals $F_{\mathrm{dyn},q},\;\;q=1,\ldots ,Q$. This can be done by applying an improved version of the Augmented Kalman Filter, as it will be shown in the following. Classic Kalman filters applied in this context [25,26] are focused on the TFRF matrix expressed in this form

(4) $$T\left(j\omega \right)=\left[\begin{array}{ccc} T_{xx}\left(j\omega \right) & T_{xy}\left(j\omega \right) & T_{xz}\left(j\omega \right)\\ T_{yx}\left(j\omega \right) & T_{yy}\left(j\omega \right) & T_{yz}\left(j\omega \right)\\ T_{zx}\left(j\omega \right) & T_{zy}\left(j\omega \right) & T_{zz}\left(j\omega \right) \end{array}\right]$$

connecting the 3×1 resultant vectors

(5) $$R_{\mathrm{dyn}}\left(j\omega \right)=T\left(j\omega \right)\;R\left(j\omega \right)$$

It is worth noting that state of the art Kalman filters for cutting force compensation are 1D or they are 3D but the cross TFRFs are usually neglected by setting $T_{ik}:=0$ (with i≠k). By adopting this simplistic approach, it is not possible to take into account the influence of input force position on dynamometer dynamics, because they are condensed into the above 3×3 transmissibility matrix.

In order to overcome this limit, a more general approach is needed. This can be achieved by focusing on the more general Q×P TFRF matrix

(6) $$W\left(j\omega \right)=\left[\begin{array}{ccc} W_{11}\left(j\omega \right) & \cdots & W_{1P}\left(j\omega \right)\\ \vdots & \ddots & \vdots \\ W_{Q1}\left(j\omega \right) & \cdots & W_{QP}\left(j\omega \right) \end{array}\right]$$

connecting the Q×1 sensed output vector $F_{\mathrm{dyn}}$ to the P×1 applied input vector F, i.e.,

(7) $$F_{\mathrm{dyn}}\left(j\omega \right)=W\left(j\omega \right)F\left(j\omega \right)$$

that may ideally incorporate all the information regarding dynamometer dynamics without any loss.

However, it was experimentally noticed that the quasi-static terms of each sensed output $F_{\mathrm{dyn},q}$ can be affected by large drifts due to thermal effects, variations of preload conditions and other unknown quasi-static disturbances. Such drifts may possibly affect the Kalman filter negatively. Nevertheless, they tend to annul each other in the linear combinations $R_{i}$ by construction (1). Accordingly, the virtual $R_{\mathrm{dyn},i}$ sensed components obtained from Equation (2) were added to the list of outputs although they are linearly dependent on their *Q* addenda. As a result, the total number of outputs was increased to $Q^{\prime }=Q+3=12+3=15$.

The state space form realization corresponding to the global transmissibility matrix (6) can be written as follows,

(8) $$\left\{\begin{array}{c} \dot{x}\left(t\right)=A\;x\left(t\right)+B\;F\left(t\right),\\ F_{\mathrm{dyn}}\left(t\right)=C\;x\left(t\right), \end{array}\right.$$

where x is the state space vector of size 4M×1 (instead of 2M×1, as it will be shortly explained), F is the applied input vector of size P×1, and $F_{\mathrm{dyn}}$ is the augmented sensed output vector of size $Q^{\prime }\times 1$.

In order to determine an accurate representation of system (8) and the associated Upgraded Augmented Kalman Filter, the following algorithm was implemented and executed.

1. Experimental modal analysis by means of pulse testing was carried out. The input hammer signal and $Q^{\prime }$ outputs were obtained from each measurement.
2. $Q^{\prime }\times P$ nonparametric, empirical TFRFs $W_{qp}\left(j\omega_{k}\right)$, coherence functions $\gamma_{qp}^{2}\left(j\omega_{k}\right)$ (k=1,2,…,N/2), and sampled Empirical Impulse Response Estimates (EIREs) $h_{qp}\left(t_{n}\right)$ with n=1,…,N were extracted from the experimental modal analysis data.
3. All the EIREs were assembled into the matrix
  (9) $$H:=\left[\begin{array}{ccc} h_{11} & \cdots & h_{1P}\\ \vdots & \ddots & \vdots \\ h_{Q^{\prime }1} & \cdots & h_{Q^{\prime }P} \end{array}\right]$$
  that has $\left(Q^{\prime }\times N\right)$ rows and *P* columns, as each $h_{qp}$ is a N×1 column vector. The Singular Value Decomposition was applied to H in order to extract the most significant $P^{\prime }=6\ll P$ principal components carrying more than 99% of the information contained into H. This allowed a considerable matrix compression that boosted the subsequent calculation.
4. The discrete Green (Hankel) matrix $H_{d}$ and the velocity related retarded Green matrix $H_{d}^{\prime }$ were assembled from the compressed EIREs matrix according to the work in [29] based on the impulse dynamic subspace (IDS) approach. The Singular Value Decomposition was applied this time to $H_{d}$ and $H_{d}^{\prime }$. By a proper multiplication of the obtained principal components the natural pulsations $\omega_{n,m}$ and the damping ratios $\xi_{m}$ of the most important m=1,…,M vibration modes were obtained. In the current case, it was necessary to include M≈50÷100 vibration modes in order to achieve a satisfactory mathematical interpolation (though probably some modes were mostly mathematical artifacts rather than physical modes). The natural frequencies and damping factors were used to construct the state space matrix A as follows,
  (10) $$A=\left[\begin{array}{cccccc} & O & & & I & \\ -\omega_{n,1}^{2} & & & -2\xi_{1}\omega_{n,1} & & \\ & \ddots & & & \ddots & \\ & & -\omega_{n,M}^{2} & & & -2\xi_{M}\omega_{n,M} \end{array}\right]$$
  where O and I are null and identity matrices of size M×M.
5. Each $W_{qp}\left(j\omega_{k}\right)$ was then expressed as the sum
  (11) $$W_{qp}\left(j\omega_{k}\right)=\frac{F_{\mathrm{dyn},q}\left(j\omega_{k}\right)}{F_{p}\left(j\omega_{k}\right)}=\sum_{m=1}^{M}\frac{G_{m,qp}+G_{m,qp}^{\prime }j\omega_{k}}{{\left(\frac{j\omega_{k}}{\omega_{n,m}}\right)}^{2}+2\xi_{m}\frac{j\omega_{k}}{\omega_{n,m}}+1},$$
  where the static gains $G_{m,qp}$ were associated to elastic force contributions due to device deformation, whereas $G_{m,qp}^{\prime }$ were associated to viscous damping forces that are proportional to the modal velocities. These coefficients were determined by stepwise linear regression and weighted linear regression procedures that were carried out in the frequency domain. As a result, a total of $\left(4MPQ^{\prime }\right)$ modal parameters have been determined so far. This would have implied a very large state space vector of size (4MP)×1, that would have caused numerical issues if not properly handled. A considerable dimension reduction was then achieved by noting that the *P* column vectors composing the matrices $G_{m,qp}$ and $G_{m,qp}^{\prime }$ (for any fixed *m*) had to be all proportional to the same eigenmode shape inspected at the $Q^{\prime }$ available outputs. The constants of proportionality were expected to be in general different from each other since they corresponded to the participation factors associated to each of the *P* inputs. However, the two matrices $G_{m,qp}$ and $G_{m,qp}^{\prime }$ had to be rank-one matrices. In other words, their first principal component obtained from SVD should have provided a good approximation of their structure, as follows,
  (12) $$G_{m}:=\left[G_{m,qp}\right]=\underset{\mathrm{from}\;\;\mathrm{SVD}}{\underset{︸}{{U\;\Sigma \;\Theta }^{H}}}\approx \underset{\mathrm{into}\;\mathrm{matrix}\;C}{\underset{︸}{\left[\begin{array}{c} u_{m,1}^{\left(1\right)}\\ \vdots \\ u_{m,Q^{\prime }}^{\left(1\right)} \end{array}\right]\sigma_{m}^{\left(1\right)}}}\underset{\mathrm{into}\;\mathrm{matrix}\;B}{\underset{︸}{\left[\begin{array}{ccc} \vartheta_{m,1}^{\left(1\right)} & \cdots & \vartheta_{m,P}^{\left(1\right)} \end{array}\right]}}$$
  and similarly for $G_{m,qp}^{\prime }$
  (13) $$G_{m}^{\prime }:=\left[G_{m,qp}^{\prime }\right]=\underset{\mathrm{into}\;\mathrm{matrix}\;B}{\underset{︸}{{{V\;S\;\Lambda }^{H}}_{\mathrm{from}\;\;\mathrm{SVD}}\approx \underset{\mathrm{into}\;\mathrm{matrix}\;C\;\;\;\;}{\underset{︸}{v_{m}^{\left(1\right)}s_{m}^{\left(1\right)}}}\underset{︸}{\lambda_{m}^{\left(1\right)}}}}$$
  Thus, only the modes satisfying this approximation were kept in the final model.
6. Then, the matrix B could be assembled as follows,
  (14) $$B=\left[\begin{array}{ccc} & O & \\ \omega_{n,1}^{2}\vartheta_{1,1}^{\left(1\right)} & \cdots & \omega_{n,1}^{2}\vartheta_{1,P}^{\left(1\right)}\\ \vdots & & \vdots \\ \omega_{n,M}^{2}\vartheta_{M,1}^{\left(1\right)} & \cdots & \omega_{n,M}^{2}\vartheta_{M,P}^{\left(1\right)}\\ & O & \\ \omega_{n,1}^{2}\lambda_{1,1}^{\left(1\right)} & \cdots & \omega_{n,1}^{2}\lambda_{1,P}^{\left(1\right)}\\ \vdots & & \vdots \\ \omega_{n,M}^{2}\lambda_{M,1}^{\left(1\right)} & \cdots & \omega_{n,M}^{2}\lambda_{M,P}^{\left(1\right)} \end{array}\right]$$
  whereas matrix C was written as follows,
  (15) $$C=\left[\begin{array}{cccccccc} u_{1,1}^{\left(1\right)}\sigma_{1}^{\left(1\right)} & \cdots & u_{M,1}^{\left(1\right)}\sigma_{M}^{\left(1\right)} & & & v_{1,1}^{\left(1\right)}s_{1}^{\left(1\right)} & & v_{M,1}^{\left(1\right)}s_{M}^{\left(1\right)}\\ \vdots & & \vdots & O & O & & & \\ u_{1,Q^{\prime }}^{\left(1\right)}\sigma_{1}^{\left(1\right)} & \cdots & u_{M,Q^{\prime }}^{\left(1\right)}\sigma_{M}^{\left(1\right)} & & & v_{1,Q^{\prime }}^{\left(1\right)}s_{1}^{\left(1\right)} & & v_{M,Q^{\prime }}^{\left(1\right)}s_{M}^{\left(1\right)} \end{array}\right]$$
  Matrix B was of size 4M×P, while matrix C was of size $Q^{\prime }\times 4M$. Accordingly, the global state space matrix A had to be doubled, i.e.,
  (16) $$A:=\left[\begin{array}{cc} A & O\\ O & A \end{array}\right]$$
  Thus, the associated state space vector was of size 4M×1, which was still large but less problematic. In detail, the final state space vector size was in the range (200÷400)×1 instead of (3200÷6400)×1.
7. In order to improve the global model adequacy, the coefficients of the B matrix were recalculated by means of another (weighted) linear regression procedure that was carried out in the frequency domain, by exploiting the relation
  (17) $$\underset{\begin{array}{l} \mathrm{known}\;\mathrm{term}\;\\ \mathrm{from}\;\mathrm{experiment} \end{array}}{\underset{︸}{W\left(j\omega_{k}\right)}}≅\underset{\mathrm{coefficient}\;\mathrm{matrix}}{\underset{︸}{C{\left(j\omega_{k}I-A\right)}^{-1}}}\underset{\mathrm{unknown}}{\underset{︸}{B}}.$$
8. The obtained dynamic system may be used for accurately estimating the measured outputs from known inputs. Nevertheless, here we were interested in the inverse problem: estimating all the inputs from the outputs. This is in general not possible when two or more distinct inputs generate very similar outputs. In order to extract the maximum amount of information regarding the input structure, the Singular Value Decomposition was applied on matrix B, thus yielding
  (18) $$B=\underset{\mathrm{from}\;\mathrm{SVD}}{\underset{︸}{U_{B}\Sigma_{B}\Theta_{B}^{H}}}\Rightarrow B\;F(t)=\underset{B_{\mathrm{pca}}}{\underset{︸}{U_{B}\Sigma_{B}}}\underset{F_{\mathrm{pca}}\left(t\right)}{\underset{︸}{\Theta_{B}^{H}F(t)}}≅{\tilde{B}}_{\mathrm{pca}}{\tilde{F}}_{\mathrm{pca}}\left(t\right),$$
  where $\tilde{□}$ denotes a reduced form including only the most important principal components. In the current approximation, $\tilde{P}=6\ll 16$ principal components were sufficient to represent ~99% of the information contained in B. Thus, the final state space form was
  (19) $$\left\{\begin{array}{l} \dot{x}\left(t\right)=A\;x(t)+{\tilde{B}}_{\mathrm{pca}}{\tilde{F}}_{\mathrm{pca}}\left(t\right),\\ F_{\mathrm{dyn}}\left(t\right)=C\;x(t), \end{array}\right.$$
  having $\tilde{P}$ “orthogonal” inputs producing distinguishable outputs, whence they could be reconstructed by applying the Kalman filter. This was a key step in order to obtain a successful filter.
9. The Upgraded Augmented Kalman Filter was obtained from the expanded state space form as
  (20) $$\left\{\begin{array}{c} {\dot{x}}_{e}\left(t\right)=\underset{A_{e}}{\underset{︸}{\left[\begin{array}{cc} A & {\tilde{B}}_{\mathrm{pca}}\\ O & O \end{array}\right]}}\underset{x_{e}\left(t\right)}{\underset{︸}{\left[\begin{array}{c} x(t)\\ {\tilde{F}}_{\mathrm{pca}}\left(t\right) \end{array}\right]}}+\underset{G_{e}}{\underset{︸}{\left[\begin{array}{c} O\\ I \end{array}\right]}}w_{proc}\left(t\right),\\ F(t)=\underset{C_{e}}{\underset{︸}{\left[\begin{array}{cc} C & O \end{array}\right]}}x_{e}\left(t\right)+w_{meas}\left(t\right), \end{array}\right.$$
  where the input force ${\tilde{F}}_{\mathrm{pca}}$ was incorporated into an expanded state space vector $x_{e}$, and it was artificially generated as the integral of a Gaussian process noise $w_{\mathrm{proc}}$ of size $\tilde{P}\times 1$. A Gaussian measurement noise $w_{\mathrm{meas}}$ of size $Q^{\prime }\times 1$ was also added to the output. O and I were null and identity matrices of adequate sizes.
10. Process and measurement noises were described by the covariance matrices
  (21) $$\left\{\begin{array}{lll} E[w_{\mathrm{proc}}{w_{\mathrm{proc}}}^{T}] & = & \left(10^{10}÷10^{12}\right)I,\\ E[{w_{\mathrm{meas}}\;w_{\mathrm{meas}}}^{T}] & = & \left(10^{0}÷10^{2}\right)I,\\ E[w_{\mathrm{proc}}{w_{\mathrm{meas}}}^{T}] & = & O, \end{array}\right.$$
  where the covariances’ orders of magnitude were selected after a preliminary trial and error phase.
11. A stationary Kalman filter was eventually determined as follows,
  (22) $$\left\{\begin{array}{c} {\dot{x}}_{e,\mathrm{obs}}\left(t\right)=\left(A_{e}-L_{e}C_{e}\right)x_{e,\mathrm{obs}}\left(t\right)+L_{e}F_{\mathrm{dyn}}\left(t\right),\\ {\tilde{F}}_{\mathrm{pca}}\left(t\right)=\left[\begin{array}{cc} O & I \end{array}\right]x_{e,\mathrm{obs}}\left(t\right), \end{array}\right.$$
  where the observer (obs) gain matrix $L_{e}$ was computed by the classical lqe Matlab routine. Finally, the inverse PCA transformation matrix was applied in order to reconstruct the input force vector, as follows,
  (23) $$F\approx {\tilde{\Theta }}_{B}{\tilde{F}}_{\mathrm{pca}}$$
  and therefore the resultant force components $R_{i}$ could be derived by applying Equation (1). It is worth noting that the Upgraded Augmented Kalman Filter (UAKF) proposed here is practically a classic Augmented Kalman Filter based on a much more general model of dynamometer dynamics.

## 4. Filter Performance Evaluation

### 4.1. Comparison with Hammer Signal during Pulse Tests

In order to assess the final performance of the proposed Kalman filter, its results were compared to those derived from state-of-the-art approaches, by considering both modal tests and cutting force data. Specifically, the force trends derived from the following four different techniques were compared,

1. raw average cutting forces obtained from the static calibration phase, see Equation (2);
2. a state-of-the-art Augmented Kalman Filter that was only based on the direct TFRFs $T_{xx}$, $T_{yy}$ and $T_{zz}$ of the global transmissibility matrix defined by Equation (4);
3. a slightly more advanced Augmented Kalman Filter that was based on the complete 3×3 transmissibility matrix of Equation (4), including cross TFRFs; and
4. the novel Upgraded Augmented Kalman Filter based on the complete $Q^{\prime }\times \tilde{P}$ model of dynamometer dynamics.

As already remarked in the previous sections, the Optimal Inverse Filtering approach [16] could not be considered here for comparison, because the data available from modal analysis were contaminated by noise in the frequency range of interest (0–10 kHz).

**Figure 4:**
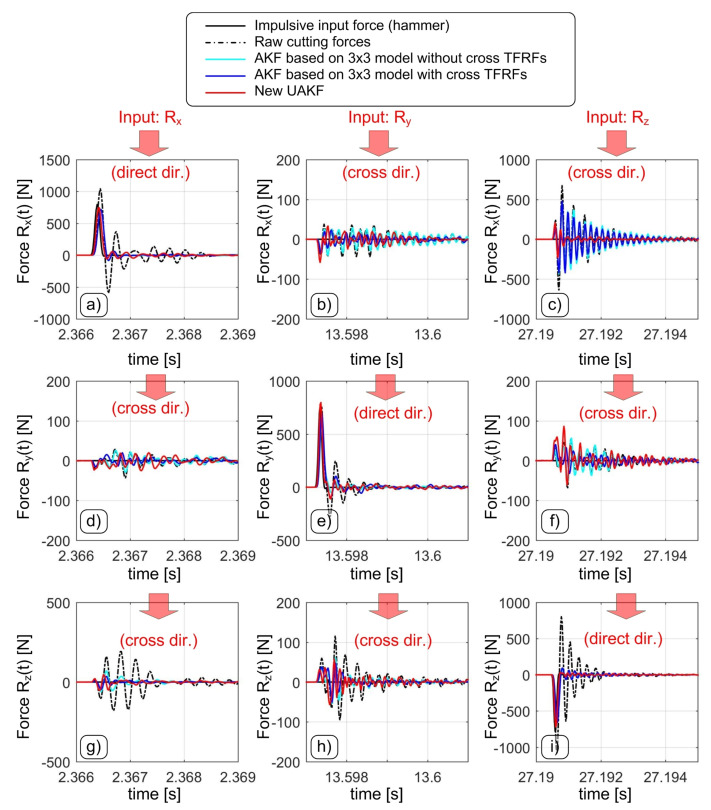
Example of generic impulse transients before and after the correction by using the developed Kalman filter. The filtered transients along the $R_{x}$, $R_{y}$ and $R_{z}$ directions when hitting the device along the $R_{x}$ direction are showed in (**a**), (**d**) and (**g**) respectively. Similarly, the filtered transients when hitting the device along the $R_{y}$ direction are showed in (**b**), (**e**) and (**h**) respectively. Eventually, the filtered transients when hitting the device along the $R_{z}$ direction are showed in (**c**), (**f**) and (**i**) respectively.

**Figure 5:**
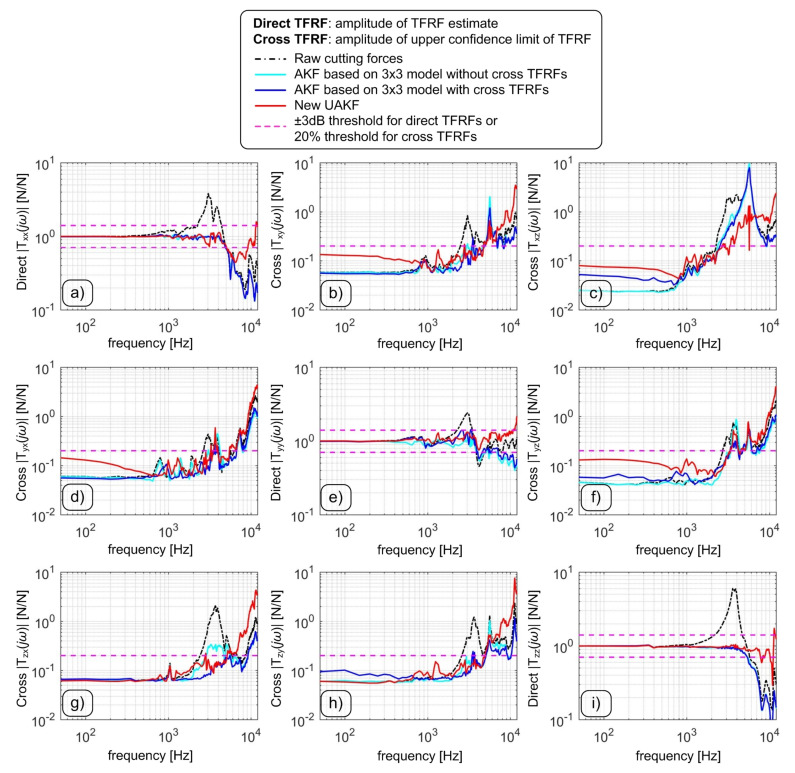
Comparison between the transmissibility frequency response functions (TFRF) compensated by the novel Kalman filter (red) and the uncompensated TFRFs (black). Confidence limits were also determined in order to include the uncertainty affecting the estimates. Subfigures (**a**–**i**) are arranged in accordance with the TFRF matrix structure of Equation (4).

The results from modal tests data in time and frequency domain are illustrated in Figure 4 and Figure 5, respectively. In general, both direct forces and crosstalk disturbances are well compensated by the Upgraded Augmented Kalman Filter. In order to quantitatively assess the achieved frequency bandwidth, the following criteria were applied,

- when considering the direct TFRFs the usable frequency bandwidth corresponds to the frequency where the TFRF amplitude exits the ±3 dB thresholds or the coherence function decreases below 80%;
- when considering cross TFRFs, the frequency bandwidth corresponds to the frequency where the upper confidence limit of the estimated cross TFRF exceeds 20%.

The final results are reported in Table 1 and Table 2.

Some fairly good results were obtained by the intermediate Augmented Kalman Filter including cross TFRFs, although the correction of some dynamic crosstalks was not satisfactory.

In detail, the filtered $R_{\mathrm{dyn},x}$ component is greatly disturbed by the forces acting along the *Z* direction. It is worth recalling that most milling operations are responsible for real 3D cutting forces. Therefore, this kind of disturbance cannot be in general avoided unless a restrictive low-pass filter is further applied to $R_{\mathrm{dyn},x}$, thus reducing its final frequency bandwidth even if the direct TFRF $T_{xx}$ is good.

On the contrary, more balanced and globally better results were obtained by the novel Upgraded Augmented Kalman Filter, that was able to compensate dynamometer dynamics up to more than 5 kHz along the main directions and up to more than 3.3 kHz along the cross, transverse directions.

In Table 2, the $r_{ii}^{2}$ symbol denotes the squared linear correlation coefficient along the *i*-direction, that was obtained by comparing the hammer signal with the force measured by dynamometer along the same direction, after the application of a given filter. Each cross-talk disturbance was characterized through the quantity $\sigma_{ik}/\sigma_{k}$ [%] given by the standard deviation of $R_{\mathrm{dyn},i}$ divided by the standard deviation of the hammer acting along the $R_{k}$ direction. It has to be noticed that only with the novel UAKF a satisfactory attenuation of the disturbance affecting the *X* direction was achieved.

### 4.2. Comparison with Cutting Force Model during Real Cutting Tests

After modal analysis, some cutting tests were also executed by using a modular Sandvik Coromant tooling system composed of a spindle adapter (C5-390B.140-40 040), an intermediate adapter (C5-391.02-32 060A), and a face shoulder cutter (R390-032C3-11M050) with external diameter D=32 mm. A single cutting insert (Z=1) Sandvik R390-11T304E-PL 1130 with nose radius $r_{\varepsilon }=0.4$ mm, lead angle $\chi_{1}=90{}^{\circ}$, and axial rake angle $\gamma_{a}\leq 15{}^{\circ}$ was mounted on the cutter in order to avoid the effects of run-out.

Down milling tests with tool–workpiece lateral immersion $a_{L}/D=50\%$ were performed according to a full factorial Design of Experiments, by varying depth of cut $a_{p}$ on 2 levels (0.4 and 0.8 mm) and feed per tooth $f_{z}$ on 2 levels (0.07 and 0.14 mm). Cutting speed $v_{c}$ was kept constant and equal to 500 m/min. This relatively high level of cutting speed was chosen to better investigate dynamometer dynamics. For each cutting parameters’ combination an entire pass was carried out, in order to evaluate the influence of cutting position on cutting force compensation. Specifically, 2 positions were selected: position 1 close to the entry side and position 2 close to the exit side, both when the cutter was completely engaged under stationary cutting conditions, see Figure 1d.

Here, a basic but state-of-the-art cutting force model was used for the sake of comparison with the filtered cutting force signals. Let $\varphi_{1}\left(t\right)$ be the feed motion angle representing the angular position of the insert, and let $g\left(\varphi_{1}\right)$ be the window function describing the tooth-workpiece engagement

(24) $$g\left(\varphi_{1}\right)=\left\{\begin{array}{cc} 1, & \mathrm{if}\;\;\;\;\varphi_{\mathrm{in}}⩽\varphi_{1}\;\;\mathrm{mod}\;\;2\pi ⩽\varphi_{\mathrm{out}},\\ 0, & \mathrm{elsewhere} \end{array}\right.$$

where $\varphi_{\mathrm{in}}=\pi /2$ was the entrance angle and $\varphi_{\mathrm{out}}=\pi$ was the exit angle under the considered down milling conditions.

As the nominal cutting edge was $\chi_{1}=90{}^{\circ}$ and the axial rake angle $\gamma_{a}$ was locally small, then the axial contributions were negligible above the nose radius. Thus, the axial terms were limited to the nose radius zone. In the light of these considerations, the following Shearing and Ploughing stationary cutting force model was adopted,

(25) $$\left\{\begin{array}{ccc} F_{1,c} & := & a_{p}\;g\left(\varphi_{1}\right)\left(k_{s,c}f_{z}\sin\varphi_{1}+k_{p,c}\right),\\ F_{1,r} & := & a_{p}\;g\left(\varphi_{1}\right)\left(k_{s,r}f_{z}\sin\varphi_{1}+k_{p,r}\right)\\ F_{1,a} & := & r_{\varepsilon }\;g\left(\varphi_{1}\right)\left(k_{s,a}f_{z}\sin\varphi_{1}+k_{p,a}\right), \end{array}\right.,\;\;\;\;\;\;\;\mathrm{with}\;\;a_{p}⩾r_{\varepsilon }$$

where $k_{s,c}$, $k_{s,r}$, and $k_{s,a}$ [N/mm${}^{2}$] are the shearing coefficients in the tangential, radial, and axial directions, respectively, whereas $k_{p,c}$, $k_{p,r}$, and $k_{p,a}$ [N/mm] are the plowing coefficients in the tangential, radial, and axial directions, respectively. This kind of model is a simplified version of well established cutting force models that can be found in literature [30,31]. Eventually, the components of the resultant force can be simply derived as follows,

(26) $$\left\{\begin{array}{lll} R_{x} & = & -F_{1,c}\cos\varphi_{1}-F_{1,r}\sin\varphi_{1},\\ R_{y} & = & F_{1,c}\sin\varphi_{1}-F_{1,r}\cos\varphi_{1},\\ R_{z} & = & F_{1,a}. \end{array}\right.$$

The six unknown cutting force model coefficients were determined by linear regression on the average cutting forces derived from the full factorial experimental design.

**Figure 6:**
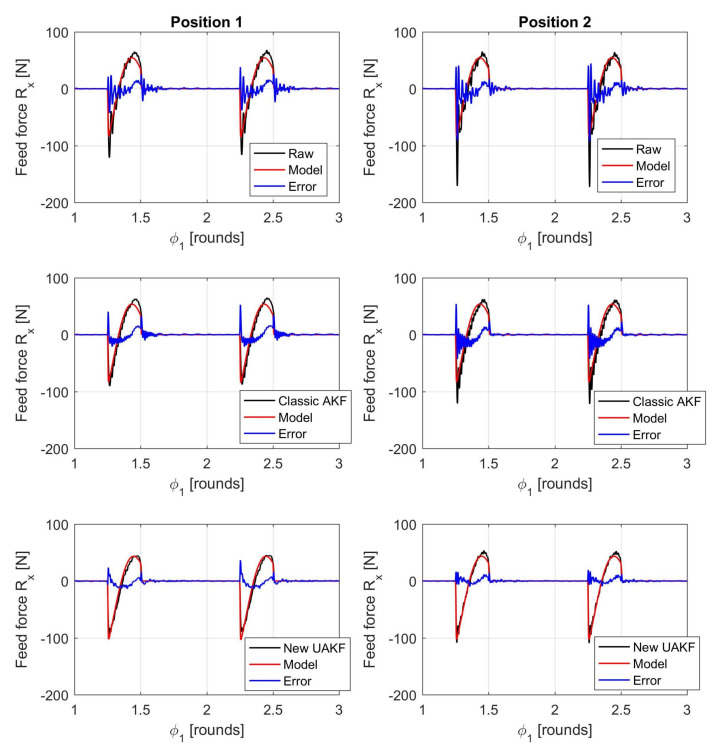
Comparison between filtered cutting forces and cutting force model along the feed direction *X* ($a_{p}=0.8$ mm, $f_{z}=0.14$ mm). The two subfigures on the top show the raw cutting forces derived from Equation (2). The two subfigures in the middle show the filtered cutting forces obtained from the classical AKF that was based on both direct and cross TFRFs. The two subfigures on the bottom show the filtered cutting forces obtained from the novel UAKF approach. Moreover, all subfigures on the left refer to position 1 (milling bass beginning) while all subfigures on the right refer to position 2 (milling pass end).

**Figure 7:**
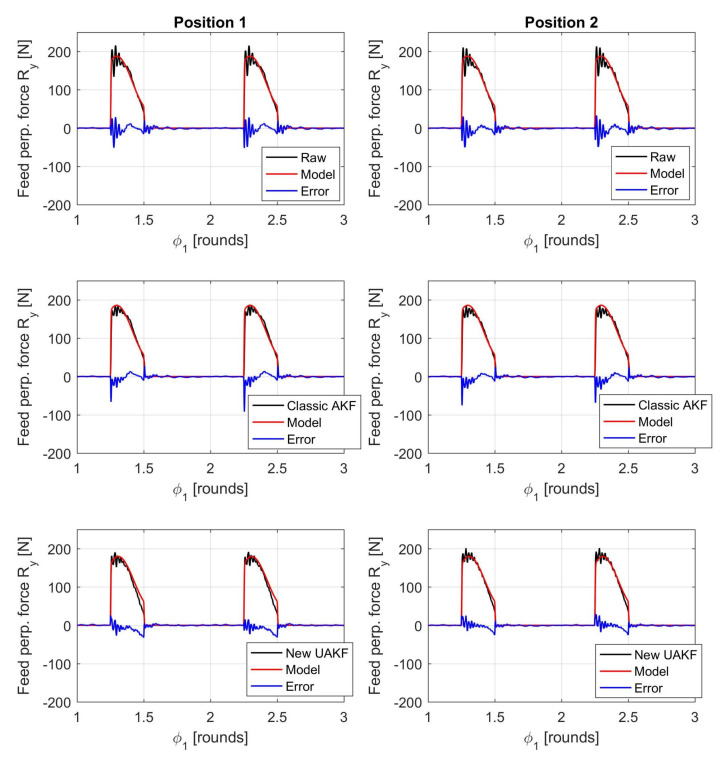
Comparison between filtered cutting forces and cutting force model along the feed perpendicular direction *Y* ($a_{p}=0.8$ mm, $f_{z}=0.14$ mm). The two subfigures in the middle show the filtered cutting forces obtained from the classical AKF that was based on both direct and cross TFRFs. The two subfigures on the bottom show the filtered cutting forces obtained from the novel UAKF approach. Moreover, all subfigures on the left refer to position 1 (milling bass beginning) while all subfigures on the right refer to position 2 (milling pass end).

**Figure 8:**
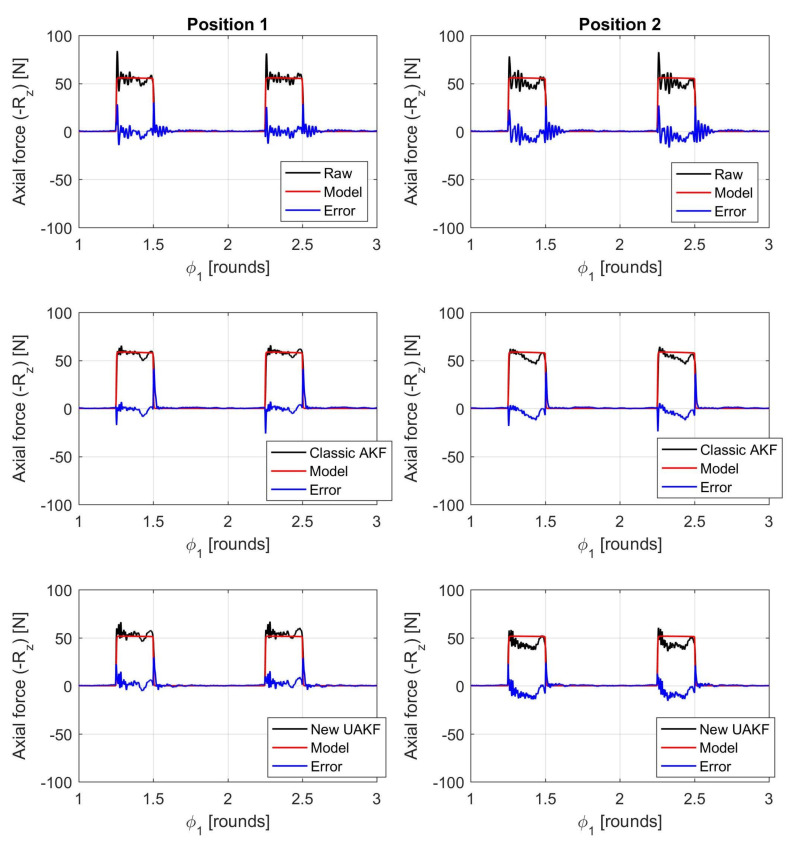
Comparison between filtered cutting forces and cutting force model along the axial direction *Z* ($a_{p}=0.8$ mm, $f_{z}=0.14$ mm). The two subfigures in the middle show the filtered cutting forces obtained from the classical AKF that was based on both direct and cross TFRFs. The two subfigures on the bottom show the filtered cutting forces obtained from the novel UAKF approach. Moreover, all subfigures on the left refer to position 1 (milling bass beginning) while all subfigures on the right refer to position 2 (milling pass end).

An example of filtered cutting forces against the interpolated cutting force model trends is illustrated in Figure 6, Figure 7 and Figure 8.

As before, $r_{ii}^{2}$ in Table 3 denotes the squared linear correlation coefficient along the *i*-direction, obtained by comparing the cutting force model trend with the force measured by dynamometer after the application of a given filter. In addition, $\sigma_{Ei}$ is the standard deviation of the absolute error between model predictions and the measured, filtered force along the $R_{i}$ direction. This parameter was normalized by the maximum absolute value along the same direction ($\max|R_{i}|$) for comparison purposes.

Visual inspection of force trends and quantitative indicators reported in Table 3 confirmed that the new Kalman filter outperforms state-of-the-art filters in terms of a full 3D cutting force correction. The higher bandwidth provided by the new filter can also be appreciated at each signal raise and decrease when the cutting insert enters and exits the workpiece (see the different error spikes at those locations). In addition, the new approach does better compensate dynamometer dynamics at both the beginning (position 1) and end (position 2) of each milling pass, contrary to the state-of-the-art AKF approach.

The reason lies in the fact that the classical AKF is based on a global oversimplified model of dynamometer dynamics that is further obtained from an averaging process. On the contrary, the novel UAKF approach is able to approximately recognize the instantaneous cutter position from the eigenmodes characteristics embedded into all the available *Q* output signals that are not immediately compressed into just three resultant force components.

## 5. Conclusions

In this work, an Upgraded Augmented Kalman Filter (UAKF) approach for a full 3D correction of cutting forces in milling was developed and successfully validated by performing experimental modal analysis and cutting tests on the device.

The modal results confirmed that the global dynamics are dominated by several vibration eigenmodes that may be differently excited depending on both the direction and application point of the input force. This last observation is generally neglected in the devoted literature, but it hinders an effective correction of the dynamic disturbances.

Here the dynamometer was considered as a complex electromechanical vibrating object showing Q=12≫3 outputs that are dynamically related to P=16≫3 different inputs. After modeling such abstract dependencies, the input–output relations could be inverted and any component of the resultant cutting force along a given direction could be retrieved as the sum of all the inputs acting along the same direction, though at different locations. The entire modeling and compensation algorithm was based on the repeated application of the Singular Value Decomposition, that played a crucial role here.

Filter performances were finally assessed by comparing the achieved frequency bandwidths along direct and cross directions. The classical Augmented Kalman Filter without cross-transmissibility frequency response functions was not able to achieve satisfactory results. Better results were obtained by a slightly more advanced filter including the cross transmissibilities, but the performance was still inadequate along one transverse direction. Globally better and more balanced results were finally attained by the novel UAKF method. Direct dynamic disturbances were compensated up to more than 5 kHz, while cross-disturbances were effectively attenuated up to 3.3 kHz.

Cutting tests were eventually executed and filtered forces were compared to a state-of-the-art cutting force model. Force signals obtained from the novel UAKF filter were closer to the well established theoretical model, at both tooth entrance and exit from the workpiece and during the tooth–workpiece engagement. A better behavior was also observed at both the beginning and at the end of each milling pass, thus showing the enhanced capacity of the filter of attenuating dynamic disturbances independently from the location where the force is applied.

In short, this method can outperform state-of-the-art methods for compensating dynamometer dynamics when its frequency response cannot be accurately identified within a broad frequency range or when cutting force signals are contaminated by noise.

However, the method is still based on a preliminary experimental modal analysis, on a complex model identification procedure and on a proper tuning of the UAKF parameters such as process and measurement noise covariances. An expert is required for all these tasks, that are far from being automatic. It would be of further interest to develop an alternative, more practical, and nonparametric method that may overcome these limitations. Some steps in this directions have been recently accomplished, e.g., by Jullien-Corrigan et al. [27], who did nevertheless not take into account the complex dynamometer dynamics illustrated here. Accordingly, further significant advances must still be done in this field.

## Figures and Tables

**Table 1:** Achieved frequency bandwidth in [kHz] by applying the different Kalman filters. *: due to coherence function decrease at that frequency.

Filter	$T_{\mathrm{xx}}$	$T_{\mathrm{yy}}$	$T_{\mathrm{zz}}$	$T_{\mathrm{xy}}$	$T_{\mathrm{xz}}$	$T_{\mathrm{yx}}$	$T_{\mathrm{yz}}$	$T_{\mathrm{zx}}$	$T_{\mathrm{zy}}$
Aver. res. components from Equation (2)	2.27	2.27	2.20	2.49	2.26	2.65	2.70	2.44	2.6
AKF 3×3 without cross terms	4.90	4.05	5.40	4.65	2.37	3.50	3.40	2.80	5.10
AKF 3×3 with cross terms	4.90	4.05	5.40	3.46	2.52	3.60	3.30	9.20	5.10
New UAKF	5.10	5.40 *	7.80	4.75	3.32	3.31	3.35	5.15	4.95

**Table 2:** Adequacy of filtered forces to the effective input forces measured by the hammer during pulse testing.

Filter	$r_{\mathrm{xx}}^{2}$	$r_{\mathrm{yy}}^{2}$	$r_{\mathrm{zz}}^{2}$	$\frac{\sigma_{\mathrm{xy}}}{\sigma_{y}}$ [%]	$\frac{\sigma_{\mathrm{xz}}}{\sigma_{z}}$ [%]	$\frac{\sigma_{\mathrm{yx}}}{\sigma_{x}}$ [%]	$\frac{\sigma_{\mathrm{yz}}}{\sigma_{z}}$ [%]	$\frac{\sigma_{\mathrm{zx}}}{\sigma_{x}}$ [%]	$\frac{\sigma_{\mathrm{zy}}}{\sigma_{y}}$ [%]
Aver. res. components from Equation (2)	0.411	0.755	0.423	19	85	12	16	45	24
AKF 3×3 without cross terms	0.466	0.764	0.867	13	68	9	15	12	9
AKF 3×3 with cross terms	0.523	0.723	0.872	9	60	7	9	6	8
New UAKF	0.868	0.835	0.873	10	19	9	12	9	9

**Table 3:** Adequacy of filtered forces to the cutting force model.

Filter	$r_{\mathrm{xx}}^{2}$	$r_{\mathrm{yy}}^{2}$	$r_{\mathrm{zz}}^{2}$	$\frac{\sigma_{Ex}}{\max|R_{x}|}$ [%]	$\frac{\sigma_{Ey}}{\max|R_{y}|}$ [%]	$\frac{\sigma_{Ez}}{\max|R_{z}|}$ [%]
Aver. res. components from Equation (2)	0.896	0.979	0.960	8.5	4.0	5.7
Kalman 3×3 without cross terms	0.892	0.980	0.974	9.4	3.9	5.3
Kalman 3×3 with cross terms	0.913	0.980	0.973	7.3	3.9	5.0
New upgraded Kalman filter	0.966	0.984	0.972	3.3	3.7	4.6

## Abbreviations

The following abbreviations and (most important) symbols are used in this manuscript.

AKF	Augmented Kalman Filter (classic from state of the art)
UAKF	Upgraded Augmented Kalman Filter (novel proposed here)
SVD	Singular Value Decomposition
PCA	Principal Component Analysis
TFRF	Transmissibility Frequency Response Function
EIRE	Empirical Impulse Response Estimate
$F_{p}$	generic input force along a given direction and at a precise location
*P*	total number of different input forces applied during modal testing
$R_{k}$	component of the resultant input force, with k=x,y,z
$F_{\mathrm{dyn},q}$	generic output signal from the dynamometer
*Q*	total number of dynamometer output signals
$R_{\mathrm{dyn},i}$	component of the resultant force derived from dynamometer, with i=x,y,z
$T_{ik}\left(j\omega \right)$	global transmissibility between $R_{k}$ and $R_{\mathrm{dyn},i}$
$W_{qp}\left(j\omega \right)$	transmissibility between $F_{\mathrm{dyn},q}$ and $F_{p}$
$Q^{\prime }$	total number of output signals including the 3 estimated resultant components
$h_{qp}\left(t_{n}\right)$	Empirical Impulse Response Estimate corresponding to $W_{qp}\left(j\omega \right)$
*N*	length of each $h_{qp}\left(t_{n}\right)$ vector
$P^{\prime }$	number of most important principal components representing input information
*M*	total number of vibration modes representing dynamometer dynamics
$G_{m,qp}$	static gain corr. to vibration mode *m* and to $W_{qp}$ caused by device deformation
$G_{m,qp}^{\prime }$	static gain corr. to vibration mode *m* and to $W_{qp}$ caused by viscous damping forces
$\omega_{n,m}$	natural pulsation of the $m^{th}$ vibration mode
$\xi_{m}$	damping ratio of the $m^{th}$ vibration mode
A, B and C	state space matrices representing full dynamometer dynamics
x	state space vector representing full dynamometer dynamics
${\tilde{B}}_{\mathrm{pca}}$	B matrix after PCA transformation
${\tilde{F}}_{\mathrm{pca}}$	input force vector after PCA transformation
$\tilde{P}$	number of most important principal components representing input information
$A_{e}$, $G_{e}$ and $C_{e}$	matrices of the expanded state space form
$x_{e}$	expanded state space vector
$w_{\mathrm{proc}}$	process noise
$w_{\mathrm{meas}}$	measurement noise
$L_{e}$	observer gain matrix
$x_{e,\mathrm{obs}}$	observed (expanded) state space vector
